# Variables associated with peripherally inserted central catheter related infection in high risk newborn infants^1^

**DOI:** 10.1590/0104-1169.3481.2488

**Published:** 2014

**Authors:** Uesliz Vianna Rangel, Saint Clair dos Santos Gomes, Ana Maria Aranha Magalhães Costa, Maria Elisabeth Lopes Moreira

**Affiliations:** 2 MSc, RN, Instituto Fernandes Figueira, Fundação Oswaldo Cruz, Rio de Janeiro, RJ, Brazil. Enfermeiro, Corpo de Bombeiros do Estado do Rio de Janeiro, Rio de Janeiro, RJ, Brazil; 3 PhD, Researcher, Instituto Fernandes Figueira, Fundação Oswaldo Cruz, Rio de Janeiro, RJ, Brazil; 4 PhD, Physician, Instituto Fernandes Figueira, Fundação Oswaldo Cruz, Rio de Janeiro, RJ, Brazil; 5 PhD, Full Professor, Instituto Fernandes Figueira, Fundação Oswaldo Cruz, Rio de Janeiro, RJ, Brazil

**Keywords:** Newborn, Catheter-Related Infections, Catheter, Biomedical Technology Assessment

## Abstract

**OBJECTIVE:**

to relate the variables from a surveillance form for intravenous devices in high risk newborn infants with peripherally inserted central catheter related infection.

**METHODOLOGY:**

approximately 15 variables were studied, being associated with peripherally inserted central catheter related infection, this being defined by blood culture results. The variables analyzed were obtained from the surveillance forms used with intravenous devices, attached to the medical records of newborn infants weighing between 500 and 1,499 g. The statistical association was defined using the Chi-squared and Student t tests. The study was approved by the Research Ethics Committee of the Instituto Fernandes Figueira under process N. 140.703/12.

**RESULTS:**

63 medical records were analyzed. The infection rate observed was 25.4%. Of the variables analyzed, only three had a statistically-significant relationship with the blood culture - the use of drugs capable of inhibiting acid secretion, post-natal steroid use, and undertaking more than one invasive procedure (p-value of 0.0141, 0.0472 and 0.0277, respectively).

**CONCLUSION:**

the absence of significance of the variables of the form may be related to the quality of the records and to the absence of standardization. It is recommended that the teams be encouraged to adhere to the protocol and fill out the form.

## Introduction

Peripherally Inserted Central Catheters (PICC) are increasingly used in Neonatal Intensive Care Units (NICU) due to their characteristics of ease of insertion and prolonged period of use. However, their use presents risks related to handling, such as mechanical complications and problems resulting from infectious processes^(^ 1 ^-^ 2 ^)^. 

In order to increase the safety of using these devices, international organizations, including the Center for Disease Control (CDC), have established protocols for the prevention of infection related to the intravascular catheter, which standardize the practice of insertion and maintenance of venous catheters, including the PICC. Adherence to the protocol requires well-trained teams and periodical monitoring of the procedures and actions defined, through standardized instruments (checklists) or surveillance forms^(^ 3 ^)^. 

In general, the checklists or the surveillance forms are developed in accordance with norms and recommendations from regulatory agencies, and are constructed based on a consensus among the professionals involved with the care processes, often without a process of validating the instrument used. In this regard, it is necessary to identify whether the items used in these documents are relatable to the outcome of infection. 

Thus, this work aims to relate the variables of the surveillance form for intravenous devices in high-risk newborn infants (NB) to infection related to the use of the catheter. 

## Methodology

In order to analyze the variables of the surveillance form related to infection due to the use of the PICC line, a transversal descriptive study was undertaken with the population of NB receiving inpatient treatment in the NICU of the National Institute of Women, Children and Adolescents' Health Fernandes Figueira, of Fiocruz (IFF/Fiocruz). This unit was selected because it has a protocol for the insertion and maintenance of PICC lines, a routine for recording procedures undertaken with the PICC by the nursing professionals in a surveillance form for intravascular devices filed in the medical records, and a technical body trained and empowered for the use of this type of protocol. The data were collected from the patients' medical records, emphasizing the surveillance forms for intravascular devices filled out by the nursing team. 

The study included all of the NB weighing between 500 and 1,499 g, born in the institution between January 2009 and December 2010, with a record of having had a PICC line in that period. NB with congenital malformations, diagnosis of infection prior to the implantation of the PICC, who were suspected of primary bloodstream infection (BSI)^(4) ^or who were transferred due to any situation were excluded from the study.

From the medical records, the researchers collected the variables related to the NB (sex, birth weight, gestational age, Apgar score, and the duration of their inpatient treatment in NICU) and to the assistance provided (ventilatory support - Continuous Positive Airways Pressure (CPAP) and mechanical ventilation); invasive procedures as umbilical catheterization, orotracheal intubation, indwelling urinary catheter, lumbar puncture, and venous resection; use of total parenteral nutrition (TPN), use of drugs for inhibiting acid secretion, post-natal steroids, use of antibiotics and blood cultures. In the surveillance form for the intravascular devices, the following variables were collected: the insertion site, the location of the distal tip of the catheter, insertion time, the indwell time, asepsis actions, dressing changes, the catheter length estimated and inserted, and the reason for its removal. The principal outcome was infection related to the use of the catheter, categorized as positive or negative according to the result of the blood culture. 

The sample size was calculated considering a prevalence of infection due to use of the PICC of 20%, a confidence level of 95%, and an error level of 10%. Based on these parameters, the minimum sample necessary for calculating the frequency of infection is 61 NB. The Student t-test was adopted for analysis of the statistical association of numerical variables, and the Chi-squared or Fisher tests were adopted for categorical variables with the infection. A level of significance of 5% was adopted for all the analyses. 

The EpiINFO 7 program was used for constructing the database, generating the tables, and the statistical tests presented. 

This work was registered with Plataforma Brasil and approved by the IFF Research Ethics Committee under number 140.703/12, as it respects the ethical standards for research involving human beings, in accordance with Resolution 196/96 of the National Health Council. 

## Results

A total of 63 medical records were analyzed, of which 53.9% (34/63) were of male NB. This population's mean birth weight was 1,105.15±235.2 grams (median of 1,115 grams), the gestational age was of 31±2.5 weeks (median of 31), Apgar at the 1^st^ minute of 1 to 9 (median of 6) and at the 5^th^ minute of 4 to 10 (median of 9) and the length of inpatient treatment was 42.5±23.1 days (median of 36.5 days). 

In relation to the care provided, it was ascertained that 81% (51/63) of the NB received some sort of ventilatory support, and that 86.2% (28/51) had records for CPAP and mechanical ventilation. Invasive procedures were observed for 100% of the NB, with 82.4% (52/63) having records of more than one invasive procedure. The variables analyzed are described in Table 1. 

**Table 1:** - Care provided to the newborn infants with Peripherally Inserted Central Catheters (PICC) in the Neonatal Intensive Care Unit of the National Institute of Women, Children and Adolescents' Health Fernandes Figueira, Rio de Janeiro, State of Rio de Janeiro (RJ), Brazil, 2009-2010 (N=63)

Care Provided	n	%
Ventilatory support	51	81.0
Continuous Positive Airways Pressure	12	23.5
Mechanical ventilation	11	21.5
Continuous Positive Airways Pressure* + *Mechanical ventilation	28	86.2
Invasive Procedures		100.0
Only one procedure	11	17.4
More than one procedure	52	82.5
TPN	63	100.0
Drugs able to inhibit acid secretion	3	4.7
Post-natal steroids	4	6.3
Antibiotics	43	68.25
Positive blood culture	16	25.40

When the blood culture was related to the use of CPAP or mechanical ventilation, significant differences were not observed (p-value of 0.924 and p-value of 0.0672, respectively). 

The variables which presented statistical significance in relation to blood culture were: more than one invasive procedure (p-value of 0.0277), use of drugs capable of inhibiting acid secretion (p-value of 0.0141), and the use of steroids in the postnatal period (p-value of 0.0472). The distribution of the values of the variables of use of drugs capable of inhibiting acid secretion and post-natal steroids, in relation to the results of the blood culture, is shown in Table 2. 

**Table 2:** - Ratio of prevalence of blood culture according to the use of drugs capable of inhibiting acid secretion and post-natal steroids, Rio de Janeiro, RJ, Brazil, 2009- 2010

Drugs	Use	Positive Blood culture	%	Prevalence	p-value
Inhibitors	Yes (n=3)	3	100.0	4.615 (2.852-7.467)	0.0141
	No (n = 60)	13	21.67		
Post-Natal Steroids	Yes (n = 4)	3	75.00	3.403 (1.620-7.148)	0.0472
	No (n = 59)	13	22.03		
Inhibitors/Post-Natal Steroids	Yes (n = 6)	4	66.67	3.000 (1.411-6.379)	0.0383
	No (n = 54)	12	22.22		

In relation to the variables from the surveillance form, the basilic vein was the most frequent PICC insertion site (41.3%), significant differences not being observed between the insertion sites and positive blood culture (p-value = 0.8977). 

The PICC insertion and indwell times were 27.2±23.7 minutes (median 26 minutes) and 10.1±10.1 days (median 9.0 days) respectively, significant differences in these times not being observed with the results of the blood cultures (Table 3).

**Table 3:** - Mean and median insertion times and indwell times for the Peripherally Inserted Central Catheter in the Neonatal Intensive Care Unit at the National Institute of Women, Children and Adolescents' Health Fernandes Figueira, Rio de Janeiro, RJ, Brazil, 2009-2010

	Blood culture	Mean	Standard-Deviation	Median	p-value
Insertion Time (min)	+	26.8	21.8	30	0.9438
	–	27.4	24.3	25	
Indwell Time (days)	+	10.69	6.322	9.5	0.6064
	–	9.88	4.87	8.5	

It was observed that 28.6% (18/63) of the medical records analyzed had records that the distal part of the PICC was inserted in the vena cava, in the 2^nd^ and 3^rd^ intercostal space. The others (71.4%) recorded the distal part of the PICC in other vessels, not identifying a statistically significant association with the results of the blood culture (p-value of 0.8977). 

It was observed that the mean length of PICC estimated to be used was 19.4±5.4 cm (median of 20 cm) and that the length effectively used was 11.0±3.3 cm (median of 10 cm). There was no significant difference between the length of PICC estimated and that effectively used, in relation to the blood culture (Table 4). 

**Table 4:** - The relationship of the Peripherally Inserted Central Catheter lengths estimated and effectively used with blood culture, in the Neonatal Intensive Care Unit at the National Institute of Women, Children and Adolescents' Health Fernandes Figueira, Rio de Janeiro, RJ, Brazil, 2009-2010

PICC length (cm)	Blood culture	Mean	Standard-Deviation	Median	p-value
Estimated to be used	+	21.3	6.3	20	0.1817
	-	18.7	5.1	20	
Effectively used	+	10.0	3.4	10	0.2287
	-	11.4	3.2	10	

**Table 5:** - The distribution of the positive blood cultures in accordance with reason for removal of the PICC in the NICU/IFF, Rio de Janeiro, RJ, Brazil, 2009 - 2010

Reason for removal	Blood culture n=57					p-value
	+	%		-	%	
End of treatment (n=36)	00	00		36	100	<0.001
Suspected, or signs of, infection of the catheter (n=16)	14	87.5		02	12.5	
Obstruction of the catheter (n=5)	02	40		03	60	

Records referring to undertaking pre-puncture aseptic procedures were found in 75% (47/63) of the forms analyzed. The products used were chlorhexidine (91%) and Povidone*-*iodine (PVP-I). 

For the variable of changing dressings, the reason for doing so recorded with the highest frequency was the occurrence of bleeding. This was not shown to be associated with positive blood culture (p-value of 0.2077). In relation to the occurrence of signs of inflammation, records were observed of induration and hyperemia at the puncture site; however, these were not shown to be statistically associated with the results of the blood culture (p-value of 1.0). 

It was observed that 100% of the PICC removed for the reason "End of treatment" did not present records for positive blood culture. Among the PICC removed due to "suspected infection of the catheter/signs of infection of the catheter", it was ascertained that 12.5% did not have records confirming the infection by blood culture (p-value < 0.001). 

## Discussion

The study assessed the relationship between the blood culture result in the variables of the surveillance form for intravascular devices for high-risk NB using PICC in a NICU. The infection rate calculated in this study was shown to be similar to that of other studies^(^ 1 ^,^ 5 ^)^. 

The association observed between the results of the blood culture and the use of drugs capable of inhibiting acid secretion (Ranitidine), post-natal steroids (Hydrocortisone and Dexamethasone) and undertaking more than one invasive procedure has already been described as a risk factor for late-onset sepsis in NB. 

In the case of ranitidine, the action inhibiting the secretion of acid from the gastric juices, a substance which has an effect on pathogenic agents, would increase the probability of infection. In relation to the use of post-natal steroids, the frequency of positive blood cultures may be explained by the action of these drugs on the immune system^(^ 6 ^-^ 7 ^)^. The simultaneity of invasive procedures was also shown to be an important factor in the predisposition to infection, which can be explained by the larger number of gateways for pathogenic agents and by the larger number of devices to be handled^(^ 4 ^)^. 

These results show the properties of these three variables in monitoring catheter related infection and the quality of its recording in the control instruments. 

The remaining variables analyzed did not show a statistically significant relationship. The principal reasons may lie in the study's transversal design, which presents limitations for temporal analyses, and in the size of the sample. Although the study respected the initial calculation of sample size for calculating the frequency of the infection rate, the analysis of the relationship of each one of the variables would require a specific sample size. For this, it would be necessary to have available data in the literature which were not found for high-risk NB, this study's population of interest. However, discussing the absence of this relationship is justified in the light of the results observed and of the theoretical framework, as when the study was initiated, it was expected that a larger number of variables contained in the surveillance form would be associated with infection. 

In relation to the location of the distal part of the PICC, previous studies have indicated that this, when inadequate, is associated with the occurrence of phlebitis, and that this event increases the risk of septicemia by approximately 18 times^(^ 8 ^)^. 

The mean indwelling time for the catheters in the group with positive blood cultures was 10.6 days. The principal reason for the removal of the PICC, and approximately 63% of the cases (36/57) was the end of the treatment. Of these, 100% did not have a positive blood culture result. This data could suggest an appropriate adherence to the protocol, however, this conclusion is limited due to the design used in the study, which observed the outcome at only one point in time. Removal due to suspected infection or signs of infection (28% - 16/57) was in accordance with that expected. The association between the PICC's indwell time and the reason for its removal allows the inference that the conducts are in conformity with the recommendations of the CDC, in item 10, category IA (strongly recommended), which stipulates the prompt removal of the device when this is no longer necessary and corroborates previous studies^(^ 3 ^,^ 9 ^-^ 11 ^)^.

In relation to the length of the PICC, the mean estimated to be inserted in the NB was 21.3 cm. However, what was observed was that only 10.0 cm were effectively inserted. This situation may represent an increased risk for infection, as the un-inserted portion of the PICC is subject to colonization through handling and through contact with the microbiota of the NB's skin, in this way possibly becoming a focus for the migration of microorganisms to the blood stream^(^ 3 ^)^.

The analysis of the pre-puncture asepsis actions evidenced chlorhexidine and PVP-I as the products used most, this last not being recommended for NB below 2,500 g, due to the transcutaneous absorption of the iodine, which can lead to hypothyroidism^(^ 12 ^)^.

## Conclusion

The quality of the records was shown to be an important stage for recovering the care history for the PICC and the surveillance of the same, pointing to the need to raise the teams' awareness regarding commitment to keeping records up-to-date, complete, and appropriately filled out. The use of standardized forms is recommended, with closed and categorized questions, periodic training, and encouragement for the teams to adhere to the protocol and to fill out the form. 

It is also recommended that further studies be undertaken covering the validation of the instruments and addressing important variables such as the use of maximum sterile barrier precautions, care taken when handling the PICC, handwashing, the time between the insertion and the appearance of infection, and the number of blood cultures taken until infection.
